# Integrated physiological, biochemical, and molecular analysis of drought tolerance in soybean cultivars

**DOI:** 10.3389/fpls.2026.1730570

**Published:** 2026-02-09

**Authors:** Fahad M. Alghabari

**Affiliations:** Department of Agriculture, Faculty of Environmental Sciences, King Abdulaziz University, Jeddah, Saudi Arabia

**Keywords:** antioxidant defense system, gene regulation, PCA, stress regulation, trait coordination

## Abstract

Drought stress is an important constraint to soybean (Glycine max L. Merr.) production, specifically in arid and semi-arid regions. This study integrated physiological, biochemical, and molecular approaches to elucidate the adaptive mechanisms of soybean genotypes against drought stress. Seven genotypes, four tolerant, ‘Ajmeri’, ‘NARC-21’, ‘DMX4561’ and ‘Rawal’, and three susceptible ‘Anjasmoro’, ‘Grobogan’, ‘Dering-1’ were evaluated under control and drought conditions in a pot experiment conducted under controlled conditions. Physiological traits, including relative water content (RWC), chlorophyll content, photosynthetic rate (Pn), and cell membrane stability (CMS), were quantified alongside biochemical indicators such as proline, glycine betaine (GB), and antioxidant enzyme activities. The relative expression of drought-responsive genes, GmDREB2, GmLEA-D11, GmP5CS, GmBADH2, GmSOD1, GmCAT1, GmPOD1, GmPIP2;9, GmCHLG was conducted via qRT-PCR. Results indicated that tolerant genotypes kept higher RWC (>80%), chlorophyll (≥1.73 g g^−1^ FW), Pn (≥30 μmol m^−2^ s^−1^), and CMS (>70%) under drought. They also accumulated more proline (up to 47.5 µg g^−1^ FW) and GB (up to 157 µg g^−1^ FW). The activities of antioxidant enzymes, in tolerant genotypes were markedly higher (CAT up to 15.3 U mg^−1^ protein; SOD >50 U mg^−1^ protein) than in susceptible genotypes. Besides, the multivariate analyses (correlation, PCA, hierarchical clustering) grouped tolerant genotypes to osmolytes (proline, GB), antioxidant enzymes, and physiological traits, proving strong drought trait association. The expression analysis showed high upregulation of stress-related genes (e.g., GmP5CS ~3.8-fold; GmBADH2 ~3.4-fold; GmSOD1 ~3.5-fold) in tolerant genotypes, rectified the physiochemical findings. Overall, these results proved that drought tolerance in soybean is regulated by the co-ordination of osmolytic adjustment, enhanced antioxidant activities, maintenance of photosynthetic traits, and transcriptional activation of stress-responsive genes. The identified tolerant genotypes will serve as promising breeding resources for the development of stress-tolerant soybean cultivars.

## 1 Introduction

Soybean (*Glycine max* L. Merr.) is a globally important legume crop valued for its high protein and oil content, as well as its role in sustainable cropping systems due to nitrogen-fixing ability. However, drought stress is a major abiotic constraint effecting soybean productivity, particularly during critical growth stages such as flowering and pod filling (Rasheed et al., 2022). With increasing climate changes, particularly in drought prone regions, the development of soybean cultivars with increased drought tolerance has become a priority for global soybean improvement programs (Wang et al., 2025).

Drought tolerance in soybean is controlled by various physiological, biochemical, and genetic factors. From a physiological perspective, traits such as relative water content (RWC), stomatal conductance (Gs), canopy wilting, chlorophyll fluorescence (Fv/Fm), and photosynthetic efficiency are widely used to assess drought performance (Singh-Bakala et al., 2025; Wang et al., 2025).

At the biochemical level, soybean plants possess various protective mechanisms in response to drought-triggered osmotic and oxidative stress. Accumulation of compatible solutes like proline and glycine betaine (GB) is a common adaptive response. Proline functions as an osmoprotectant, stabilizer of various proteins and membranes, and scavenger of reactive oxygen species (ROS), helping to protect cells under dehydration (Alzahrani, 2024). Besides, GB, another well-documented osmoprotectant, protects thylakoid membranes and enzymes, and maintains photosynthetic activity under water-deficit conditions (Liu et al., 2017).

Drought also triggers the generation of ROS, causing oxidative stress and significant damage to lipids, proteins, and nucleic acids. The activation of antioxidant defense enzymes, such as superoxide dismutase (SOD), catalase (CAT), and peroxidase (POD) is important in mitigating this oxidative damage caused by the drought stress (Hasanuzzaman et al., 2020). The extent of lipid-peroxidation is often measured by osmoprotectants, which serves as a biochemical marker for cell membrane stability (CMS). Drought-tolerant soybean genotypes generally show enhanced accumulation of osmolytes and higher CMS that indicates better cellular protection under stress (Nazar et al., 2012).

Osmoprotectant accumulation and efficient ROS scavenging are essential components of drought tolerance in soybean. Enhanced levels of proline and GB contribute to osmotic adjustment and membrane protection, while increased activities of antioxidant enzymes such as CAT, SOD and POD mitigate drought-induced oxidative damage in tolerant soybean genotypes (Alzahrani, 2024). Recent integrative physiological and transcriptomic studies further demonstrate that coordinated regulation of osmolyte metabolism and antioxidant defense pathways collectively determines the drought resilience in soybean (Liu et al., 2017), hence supporting their use as reliable selection traits in soybean breeding programs.

Genetically, drought-tolerance in soybean is mediated by a set of stress-responsive genes controlled by various transcription factors. For instance, *GmP5CS* encodes Δ¹-pyrroline-5-carboxylate synthetase, a key enzyme in proline biosynthesis, and its increased expression enhances proline accumulation for osmotic adjustment under drought stress (Soleimani et al., 2015). Moreover, *GmDREB2* is a dehydration-responsive element-binding transcription factor that regulates multiple drought-inducible genes involved in stress adaptation (Pham et al., 2020). Together, these genes contribute to improved drought tolerance in soybean by coordinating osmolyte accumulation and stress-responsive signaling pathways. Moreover, *GmSOD1, GmPOD1*, and *GmCAT1* encode key antioxidant enzymes, SOD, POD, and CAT, that function in coordination to detoxify drought-induced ROS, thereby protecting cellular membranes and macromolecules in soybean under water deficit conditions (Easwar Rao and Viswanatha Chaitanya, 2020; Alzahrani, 2024). The gene *GmBADH*, encoding betaine aldehyde dehydrogenase, plays an eminent role in GB biosynthesis, contributing to osmotic adjustment and stabilization of proteins and membranes during drought and salinity stress in soybean (Liu et al., 2017). In addition, *GmCHLG*, encoding chlorophyll synthase, regulates chlorophyll biosynthesis and is critical for maintaining chlorophyll content and photosynthetic capacity under drought stress (Zhang et al., 2018). Besides, *GmPIP2;9* encodes a plasma membrane aquaporin that facilitates water transport across cell membranes, thereby contributing to improved water uptake and maintenance of cellular water balance under drought stress in soybean (Lu et al., 2018). In parallel, *GmLEA-D11* encodes a late embryogenesis abundant (LEA) protein that protects cellular proteins and membranes from dehydration-induced damage (Savitri, 2023). The coordinated expression of these genes enhances drought tolerance in soybean by improving water-use efficiency and maintaining cellular stability under water-limited conditions. Collectively, these genes contribute to soybean drought tolerance through the integrated regulation of ROS scavenging, osmotic protection, and physiological stability, highlighting their relevance as molecular targets for drought-tolerance screening and breeding (Liu et al., 2017).

Although sufficient research has been done, most studies have evaluated these traits individually, but there is a growing need to provide integrated physiological, biochemical, and genetic insights to better understand the complicated nature of drought-tolerance in soybean for developing more efficient selection strategies. Therefore, this study aimed to optimize the selection of soybean cultivars under drought stress by comprehensively evaluating the physiological, biochemical and genetic responses of drought tolerance in soybean. Furthermore, to explain the drought tolerance in soybean by the combined regulation of plant water status (RWC), photosynthetic performance, membrane stability, antioxidant defense, and stress-responsive gene expression rather than by any single indicator. This integrated approach will help to identify superior cultivar-trait combinations and will support the development of soybean varieties better adapted to drought-prone environments.

## 2 Materials and methods

### 2.1 Plant material and experimental design

In this study, drought susceptible and tolerant soybean genotypes were obtained from different research institutions (**Table 1**), were evaluated for their responses to drought stress using physiological, biochemical, and molecular approaches. A two factorial experiment with three replications was conducted in a completely randomized block design (RCBD), with genotype (first factor) and treatment (well-watered and drought stress) as the second factor. The pot trial was set up at the research station of King Abdulaziz University, Jeddah, Saudi Arabia (21°32′36″N, 39°10′22″E; 12 meters above sea level).

**Table 1:** List of soybean genotypes used in study.

Genotype	Origin/Institute	Release year	Reference
Drought Tolerant			
Ajmeri	National Agricultural Research Centre (NARC), Islamabad, Pakistan	~1980s – 1990s	Mehmood et al., 2023
NARC-21	National Agricultural Research Centre (NARC), Islamabad, Pakistan	Not officially documented (> 2010)	Alzahrani, 2024
Rawal (= Rawal-1)	National Agricultural Research Centre (NARC) and Pakistan Agricultural Research Council (PARC), Islamabad, Pakistan	1993	Manzoor et al., 2022
DMX4561 (DM45X61)	Argentina/USA, Donmario (GDM Seeds)	Breeding line (post-2018 use)	Alzahrani, 2024
Drought Susceptible			
Anjasmoro	Indonesian Legumes and Tuber Crops Research Institute (ILETRI) (Balitkabi), Malang, Indonesia.	2001	Romeida et al., 2024
Grobogan	Indonesian Legumes and Tuber Crops Research Institute (ILETRI) (Balitkabi), Malang, Indonesia.	2008	Romeida et al., 2024
Dering-1	Indonesian Legumes and Tuber Crops Research Institute (ILETRI) (Balitkabi), Malang, Indonesia.	2012	Romeida et al., 2024

### 2.2 Plant cultivation, stress application and data collection

Seeds of *Glycine max* (soybean) cultivars were surface-disinfected using 2% sodium hypochlorite for 3 min, followed by thorough rinsing with double distilled water. Sterilized seeds were sown in 25 cm diameter plastic pots filled with 2:1:1 (v/v) mixture of loam soil, sand, and well-decomposed farmyard manure to facilitate sufficient aeration and drainage. Pots were grown under controlled greenhouse conditions and irrigated regularly to facilitate uniform germination and seedling establishment. Two plants per pot were retained and for each treatment five pots were used. The glasshouse temperature was adjusted at 28 ± 2°C during the day, and 22 ± 2°C at night, with a relative humidity level of 60–70% and a 14 h light/10 h dark photoperiod following Makbul et al. (2011). The experiment was continued for 45 days after sowing, with the drought stress period lasting 15 days, which induced measurable stress responses without causing irreversible damage. Besides, soybean plants were maintained under well-watered conditions until the V_3_ vegetative stage. Afterward, the drought stress was imposed by withholding irrigation or maintaining soil moisture at 30–40% field capacity (Board and Kahlon, 2011). Soil moisture at 30–40% field capacity was measured using the gravimeter. Field capacity at 100% was measured after saturating the soil and allowing free drainage and oven-drying a subsample at 105°C for 24 h. The required moisture level was maintained by weighing the pot. Furthermore, soil moisture at 30–40% of field capacity was calculated as 30–40% of the water content at 100% field capacity. It was maintained throughout the experiment and the measurements for FC were taken using the following equation,


FC=(Wf−Wd/Wd)×100,whereas Wf​=fresh soil weight and Wd one=oven−dry soil weight.

For physiological, biochemical and genetic comparisons of genotypes the measurements were taken at once, at the end of the drought stress period, when stress responses were fully expressed before permanent wilting (Makbul et al., 2011). For each treatment the data were taken from three random pots, and averaged before analysis. Besides, the effect of positional error was minimized by changing the positions of pots,

### 2.3 Antioxidant enzyme assays and osmoprotectant quantification

Activities of catalase (CAT), peroxidase (POD), and superoxide dismutase (SOD) were calculated spectrophotometrically following the protocol of Alici and Arabaci (2016). Fresh soybean leaf samples were rinsed, frozen at −20°C, and homogenized in 100 mM sodium phosphate buffer (pH 7.0) having 1 mM ascorbic acid and 0.5% (w/v) PVP. Afterward, the homogenate was incubated at 4°C for 5 min, filtered through muslin cloth, and centrifuged at 5000 × g for 5 min to attain the supernatant as the enzyme extract. The CAT activity from extract was quantified by monitoring the decline in absorbance at 240 nm due to H_2_O_2_ decomposition (Chance and Maehly, 1955). Besides, POD activity was assessed via the increase in absorbance at 420 nm using 4-methylcatechol as substrate (Kar and Mishra, 1976). Moreover, the SOD activity was measured based on inhibition of NBT photoreduction, with absorbance read at 560 nm (Beauchamp and Fridovich, 1971). The enzyme activities were expressed in standard enzyme units. Proline content was quantified following the acid ninhydrin method described by Bates et al. (1973), in which fresh leaf tissues were homogenized in 3% sulfosalicylic acid, reacted with acid ninhydrin and glacial acetic acid. Subsequently heated at 100°C for 1 h, and the chromophore was extracted with toluene before measuring absorbance at 520 nm. Moreover, Glycine betaine (GB) was measured following the protocol of Grieve and Grattan (1983), where plant material dried in oven was extracted in deionized water. Afterward, treated with iodine–potassium iodide reagent and subjected to incubation at 4°C until precipitate formation. The precipitate was dissolved in 1,2-dichloroethane to record the absorbance at 365 nm.

### 2.4 Physiological parameters

Photosynthetic rate (Pn) was calculated by a portable Infrared Gas Analyzer (IRGA; ADC Bioscientific, UK). The readings for Pn were taken between 09:00 and 11:00 am to minimize diurnal variation. Fully expanded upper canopy leaves were clamped in the leaf chamber with photosynthetically active radiation (PAR) ranged approximately 800–1200 μmol m^−2^ s^−1^, leaf temperature maintained at 25–30°C, and ambient CO_2_ concentration at approximately 380–400 μmol mol^−1^. Besides, relative humidity followed natural conditions, and data were taken when gas-exchange parameters stabilized to ensure accurate CO_2_ assimilation values. Furthermore, total chlorophyll was determined following Wellburn (1994) method. Fresh leaf tissue (0.2 g) was extracted in 10 mL of 80% acetone and centrifuged at 10,000 rpm for 10 mins. Besides, spectrophotometric absorbance was recorded at 645 and 663 nm using 80% acetone as blank. Besides, total chlorophyll (mg L^−1^) was calculated as (20.2 × A645 + 8.02 × A663), and expressed as g kg^−1^ of fresh weight using the formula: total chlorophyll (g kg^−1^ FW) = (mg L^−1^ × extract volume in liters)/fresh weight in kilograms. Cell membrane stability percentage (CMSP) was calculated using the electrolyte leakage method described by Ibrahim and Quick (2001). Leaf discs were incubated in deionized water and initial electrical conductivity E1 was measured, followed by autoclaving to release total electrolytes to record final electrical conductivity E2. CMSP was calculated based on the ratio of initial to final conductivity values using the formula, [CMSP = 1−(E1/E2) ×100]. Relative water content (RWC) was measured following the protocol of Barrs and Weatherley (1962). Fresh leaf samples were weighed immediately, rehydrated to full turgidity, and then oven-dried to constant weight. RWC was calculated using fresh (FW), turgid (TW), and dry weights (DW) as


RWC (%)=(FW−DW/TW−DW)×100

### 2.5 RNA isolation and gene expression analysis

Leaf samples for transcript analysis were collected at the onset of drought symptoms, including wilting, leaf rolling, turgidity loss, slight drooping of upper leaves, and a dull green to pale-coloration as compared with well-watered control plants. Besides, the sampling was performed at the initiation of early stress symptoms to capture initial transcriptional responses before the severe stress-induced damages. Collected leaf samples from soybean for transcript analysis were immediately frozen in liquid nitrogen to store at −80°C. Besides, total RNA was extracted using the RNeasy Plant Mini Kit (Qiagen, Germany) and reverse-transcribed from 2 µg RNA using the QuantiTect Reverse Transcription Kit (Qiagen, Germany). Furthermore, Quantitative real-time PCR (qRT-PCR) was conducted using SYBR Green PCR Master Mix (BioFACT, Korea) to quantify the expression of drought-responsive genes, with *GmActin1* as the internal reference. All reactions were executed in triplicate (biological and technical), and expression levels were calculated using the 2^−ΔΔCt method (Chen et al., 2013). The list of primers used is indicated in **Table 2**.

**Table 2:** List of gene primers used for relative gene expression analysis.

Gene	Forward primer (5′→3′)	Reverse primer (5′→3′)	Reference
GmDREB2	ATGGAAGAAGCGTTAGGTGGAGA	TGGAGGACGTCGAGTATTGTGG	Chen et al., 2022
GmLEA-D11	ATGATCAGGGTCGCAAGGTC	CTTGTCACTGTGTCCTCCAG	Guo et al., 2013
GmP5CS	CCTGTTGCTGGTGCTTCTTC	GGTTGAGGTCCTTGGAGTGA	Soleimani et al., 2015
GmSOD1	TTGCTGGTCTTAAGCCTGGT	GGCAACCATTGGTAGTGTCC	Imran et al., 2021
GmPOD1	ACATTGGAGTGCTAACGGGA	TGAGCTAACCATGCCATCTGA	Imran et al., 2021
GmCAT1	GAACAACTTCAAGCAGCCCG	GCCTCGTGCTGAGATGAGAA	Imran et al., 2021
GmBADH2	GCTGAATGGACCATATTTGGTTGC	GAGAGCAATTAATCCACACAATTCCAGC	Qian et al., 2022
GmPIP2;9	TCACTTGGCAACCATCCCAG	CAAGAGCCTTAGCAGCACCT	Lu et al., 2018
GmCHLG	GCTTCACCGTTCTCCTCTTC	GGTAGCAGCAGCAGTTCTTC	La et al., 2022

### 2.6 Statistical analysis

The experiment was performed in RCBD with two factorial arrangement of treatments. Two-way ANOVA was performed using Statistix version 8.1 (McGraw-Hill, 2008) at a 5% probability level to assess the effects of treatments, water regimes (control and drought) and genotypes on the measured traits. Each treatment combination was replicated three times (blocks). When ANOVA indicated significant differences, means were separated using the Least Significant Difference (LSD) test at p≤ 0.05. Besides, prior to analysis, the assumptions of normality and homogeneity of variance were examined.

Moreover, RStudio version 1.1.456 (RStudio Team, 2020) was used to establish correlogram, heatmap clusters and principal component analysis (PCA) biplot. The R packages “factoextra” and “FactoMineR” were used to establish the PCA biplot. Besides, the heatmap cluster dendrogram was constructed using the R packages, “pheatmap” and “complex Heatmap”. The R packages “GGally” and “ggplot2” were used to execute Pearson correlation.

## 3 Results

### 3.1 Physiological traits

**Figure 1:**
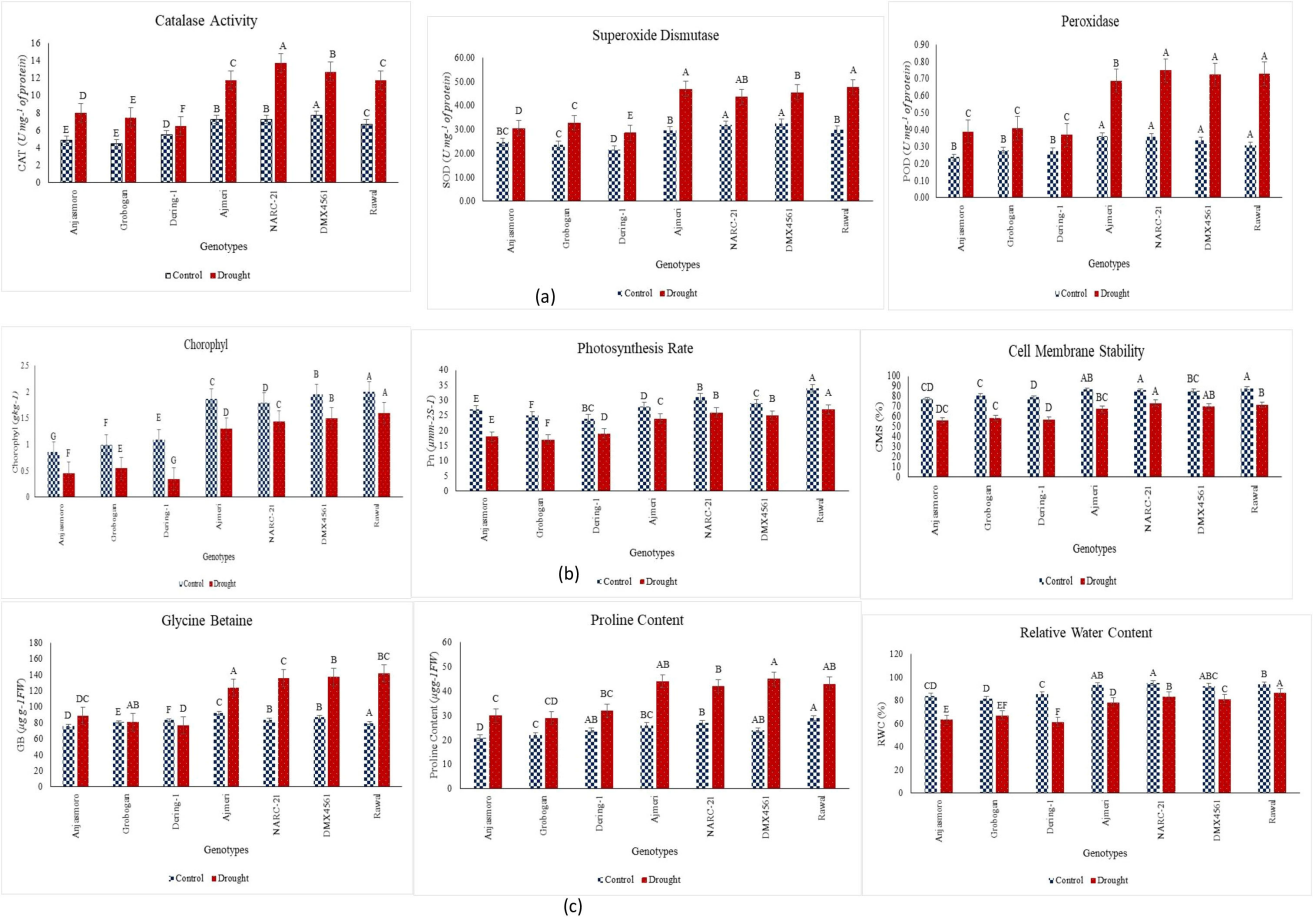
Physiological and biochemical responses of drought tolerant (Ajmeri, NARC-21, DMX4561, and Rawal) and drought susceptible (Anjasmoro, Grobogan, and Dering-1) soybean genotypes under control and drought treatments: **(a)** comparative activities of catalase (CAT), superoxide dismutase (SOD), and peroxidase (POD); **(b)** chlorophyll content, photosynthetic rate (Pn), and cell membrane stability (CMS); **(c)** glycine betaine (GB), proline content, and relative water content (RWC) across seven soybean genotypes. Data represent means ± SE (n = 3). Different letters above the bars indicate significant differences among genotypes at p ≤ 0.05 (LSD).

Drought stress significantly (p ≤ 0.05) reduced chlorophyll content, photosynthetic rate (Pn), and cell membrane stability (CMS) across all soybean genotypes, with pronounced genotypic variation as evident in **Figure 1**. Under drought conditions, chlorophyll content was the lowest in susceptible genotypes ‘Anjasmoro’, ‘Grobogan’, and ‘Dering-1’, remaining below 0.6 g kg^−1^ (**Figure 1**). In contrast, tolerant genotypes maintained comparatively higher chlorophyll levels, with ‘Ajmeri’, ‘NARC-21’, ‘DMX4561’, and ‘Rawal’ showing drought values ranging from about 1.3 to 1.6 g kg^−1^, and ‘Rawal’ recording the highest chlorophyll content under stress (**Figure 1**).

Photosynthetic rate (Pn) followed a corresponding pattern. Drought stress caused a noteworthy decline in Pn among all genotypes; however, susceptible genotypes such as ‘Anjasmoro’ and ‘Grobogan’ exhibited the lowest rates under drought (approximately 17–18 μmol m^−2^ s^−1^) as shown in **Figure 1**. Conversely, the tolerant genotypes kept higher Pn activity, with ‘Ajmeri’, ‘NARC-21’, ‘DMX4561’, and ‘Rawal’ maintaining Pn values approximately between 24 and 28 μmol m^−2^ s^−1^, and ‘Rawal’ showing the highest Pn under drought conditions (**Figure 1**).

Cell membrane stability was also significantly affected by drought stress. CMS values declined across all genotypes, but susceptible genotypes (‘Anjasmoro’, ‘Grobogan’, and ‘Dering-1’) exhibited the lowest CMS under drought, remaining close to 55–60%. In contrast, tolerant genotypes maintained higher CMS values, with ‘Ajmeri’, ‘NARC-21’, ‘DMX4561’, and ‘Rawal’ showing drought CMS values of approximately 68–75%, and ‘NARC-21’ and ‘Rawal’ ranking highest among the genotypes (**Figure 1**). Overall, **Figure 1** demonstrates that genotypes with greater drought tolerance consistently maintained higher chlorophyll content, Pn, and CMS under water-limited conditions.

### 3.2 Osmoprotectants and relative water content

As shown in **Figure 1**, drought stress significantly (p ≤ 0.05) induced the accumulation of osmoprotectants, glycine betaine (GB) and proline alongside a variable decline in relative water content (RWC) across all soybean genotypes. GB content increased significantly (p≤ 0.05) under drought stress in all genotypes (**Figure 1**). Under drought conditions, GB content ranged approximately from 85 to 145 µg g^−1^ FW. The lowest GB accumulation was observed in ‘Grobogan’ (approximately 80–85 µg g^−1^ FW) and **‘**Dering-1 (approximately 75–80 µg g^−1^ FW), whereas ‘Anjasmoro’ showed a moderate increase (90 µg g^−1^ FW) as shown in **Figure 1**. In contrast, drought-tolerant genotypes accumulated substantially higher GB levels, with ‘Ajmeri’ (125 µg g^−1^ FW)**, ‘**NARC-21’ (135 µg g^−1^ FW), DMX-4561 (140 µg g^−1^ FW), and ‘Rawal’ (145 µg g^−1^ FW) showing the highest accumulation (**Figure 1**).

Proline content also increased significantly (p≤ 0.05) under drought stress among all genotypes (**Figure 1**). Under drought conditions, proline content ranged approximately from 30 to 47 µg g^−1^ FW (**Figure 1**). The lowest proline accumulation was recorded in ‘Anjasmoro’ (0 µg g^−1^ FW) and ‘Grobogan’ (approximately 28–30 µg g^−1^ FW), followed by ‘Dering-1’ (32 µg g^−1^ FW). Higher proline accumulation was observed in ‘NARC-21’ (42 µg g^−1^ FW) and ‘Ajmeri’ (44 µg g^−1^ FW), while the maximum proline content was recorded in ‘DMX-4561’ (approximately 46–47 µg g^−1^ FW) and ‘Rawal’ (approximately 43–45 µg g^−1^ FW) as indicated in **Figure 1**. The letter groupings indicate significant genotypic differences under drought stress (LSD, p≤ 0.05).

Besides, RWC was substantially maintained in tolerant genotypes, with ‘Rawal’, ‘Ajmeri’, and ‘NARC-21’ exhibiting values >80% under drought. In contrast, ‘Anjasmoro’ and ‘Grobogan’ showed significant water loss (RWC< 65%) (**Figure 1**). These results demonstrate that the synthesis of compatible solutes and water retention are critical components of drought adaptation in soybean.

### 3.3 Antioxidant enzymes

The activities of antioxidant enzymes catalase (CAT), superoxide dismutase (SOD), and peroxidase (POD) showed significant (p ≤ 0.05) genotypic variation under drought stress (**Figure 1**). Drought-tolerant genotypes exhibited a stronger enzymatic response compared to susceptible ones. Catalase activity was markedly higher in ‘NARC-21’ (15.3 U/mg protein) and ‘DMX4561’ (13.1 U/mg protein), followed closely by ‘Rawal’ and ‘Ajmeri’, indicating effective ROS scavenging under stress (**Figure 1**). In contrast, susceptible genotypes ‘Anjasmoro’, ‘Grobogan’, and ‘Dering-1’ maintained significantly lower CAT activity (<9 U/mg protein) (**Figure 1**). A similar trend was observed for SOD, where ‘Rawal’ and ‘Ajmeri’ recorded values above 50 U/mg protein, whereas ‘Grobogan’ and ‘Dering-1’ showed values below 40 U/mg (**Figure 1**). The POD activity increased significantly (p≤ 0.05) in all genotypes under drought, but the most pronounced elevation was seen in ‘NARC-21’, ‘Rawal’, ‘Ajmeri’, and ‘DMX4561’ (0.70-0.80 U/mg protein), whereas ‘Anjasmoro’, ‘Dering-1’ and ‘Grobogan’ exhibited limited increases (0.40-0.45 U/mg protein) (**Figure 1**). These results demonstrate that enhanced antioxidant activity in tolerant genotypes contributes to their superior drought resilience by mitigating oxidative stress.

### 3.4 Gene expression analysis

**Figure 2:**
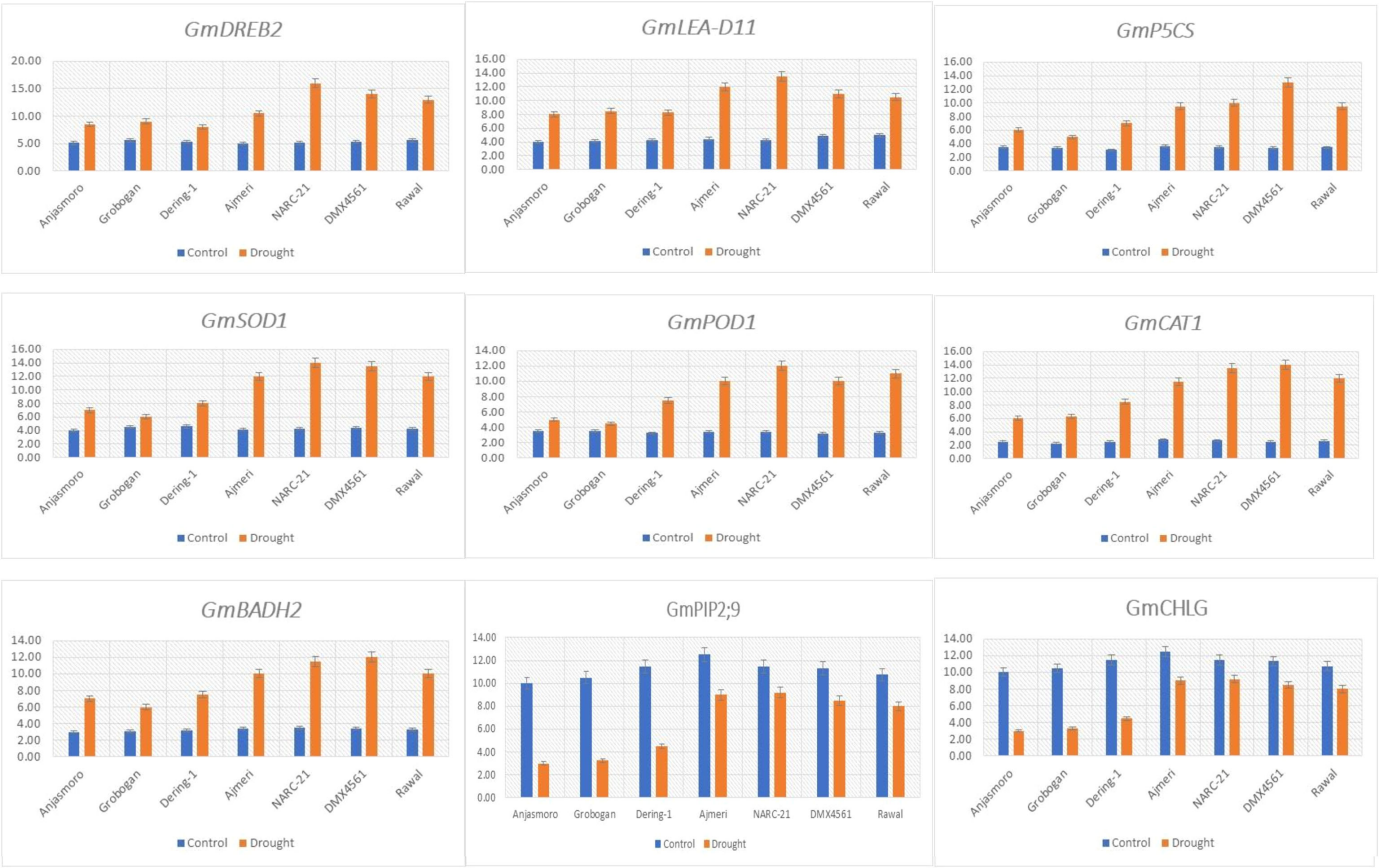
Relative expression patterns of drought associated genes in drought tolerant (Ajmeri, NARC-21, DMX4561, and Rawal) and drought susceptible (Anjasmoro, Grobogan, and Dering-1) soybean genotypes under control and drought conditions. Quantitative gene expression levels (mean ± SE, n = 3) of nine drought responsive genes: GmDREB2, GmLEA-D11, GmP5CS, GmSOD1, GmPOD1, GmCAT1, GmBADH2, GmPIP2;9, and GmCHLG.

The expression profiles of drought-responsive genes revealed a consistent difference between tolerant (‘Ajmeri’, ‘NARC-21’, ‘DMX4561’, ‘Rawal’) and susceptible (‘Anjasmoro’, ‘Grobogan’, ‘Dering-1’) soybean genotypes as shown in **Figure 2**. The transcription factor gene, *GmDREB2* showed higher expression in tolerant genotypes, with fold increases ranging from ~2.0 to 3.3-fold relative to control, compared to ~1.8 to 2.0-fold in susceptible genotypes, indicating stronger activation of downstream stress defense pathways (**Figure 2**). Moreover, *GmLEA-D11*, associated with dehydration tolerance, also displayed greater upregulation in tolerant genotypes (~2.8 to 3.6-fold) than in susceptible ones (~1.7 to 2.1-fold) as indicated in **Figure 2**. The proline biosynthesis gene *GmP5CS* exhibited markedly higher expression (**Figure 2**) in tolerant genotypes (~2.9 to 3.8-fold) than in susceptible genotypes (~1.8 to 2.2-fold), closely reflecting the higher proline accumulation observed in biochemical analyses (**Figure 1**). Genes encoding antioxidant enzymes, *GmSOD1*, *GmPOD1*, and *GmCAT1*, also followed corresponding trend, with tolerant genotypes showing fold increases of ~2.7 to 3.5-fold compared to ~1.6 to 2.0-fold in susceptible genotypes (**Figure 2**), consistent with the elevated enzymatic activities recorded in **Figure 1**. The osmolyte biosynthesis gene *GmBADH2*, responsible for GB production, was more strongly expressed in tolerant genotypes (~2.8 to 3.4-fold) than in susceptible genotypes (~1.7 to 2.0-fold) (**Figure 2**), aligning with their higher GB content observed in **Figure 1**. For water transport, *GmPIP2;9* expression was maintained or slightly increased in tolerant genotypes (~1.3 to 1.6-fold) but declined in susceptible ones (~0.8 to 1.0-fold) as indicated in **Figure 2**, indicating reduced aquaporin-mediated water movement in sensitive backgrounds. The chlorophyll biosynthesis gene *GmCHLG* was downregulated in all genotypes under drought but remained higher in tolerant lines (~0.8 to 1.0-fold) compared to susceptible lines (~0.5 to 0.7-fold) (**Figure 2**), corresponding to their better chlorophyll retention (**Figure 1**). These consistent fold-change patterns across genes reinforce the earlier physiological, biochemical, and statistical findings (**Figure 1**), demonstrating that drought tolerance in soybean is regulated by coordinated transcriptional activation of genes involved in stress signaling, osmotic adjustment, antioxidant defense, water transport, and chlorophyll biosynthesis.

### 3.5 Statistical interpretations

**Figure 3:**
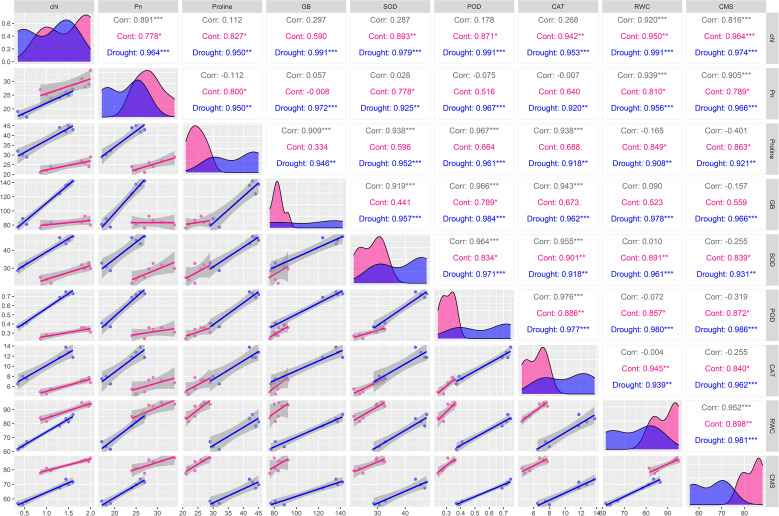
Correlation matrix of physiological and biochemical traits of drought tolerant (Ajmeri, NARC-21, DMX4561, and Rawal) and drought susceptible (Anjasmoro, Grobogan, and Dering-1) soybean genotypes under control and drought treatments. Pearson’s correlation coefficients are shown for chlorophyll (chl), photosynthetic rate (Pn), proline, glycine betaine (GB), superoxide dismutase (SOD), peroxidase (POD), catalase (CAT), relative water content (RWC), and cell membrane stability (CMS). Pink and blue represent control and drought conditions, respectively. Asterisks denote significance levels (p< 0.05, *p< 0.01, **p< 0.001, ***p≤ 0.001).

**Figure 4:**
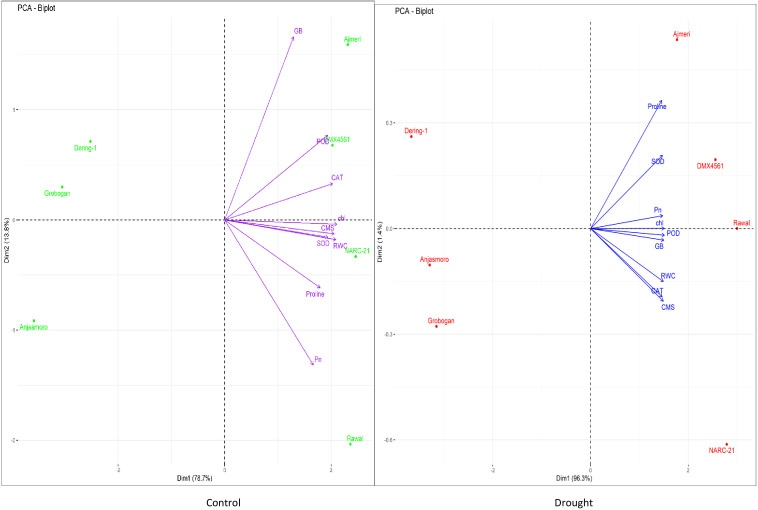
Principal component analysis (PCA) biplots of drought tolerant (Ajmeri, NARC-21, DMX4561, and Rawal) and drought susceptible (Anjasmoro, Grobogan, and Dering-1) soybean genotypes under control and drought treatments. Biplots represent trait loadings and genotype distribution under (left) control and (right) drought conditions. Variables include proline, glycine betaine (GB), superoxide dismutase (SOD), peroxidase (POD), catalase (CAT), chlorophyll (chl), photosynthetic rate (Pn), relative water content (RWC), and cell membrane stability (CMS). Genotypes clustering near trait vectors show strong positive associations with those traits.

The multivariate statistical analysis provided further validation and deeper insights into the physiological and biochemical responses of the soybean genotypes under drought stress. The correlation matrix and pairwise scatter plots in **Figure 3** revealed highly significant positive associations among drought-responsive traits. Under drought conditions, strong correlations were observed between proline and GB (r = 0.991***), as well as between proline and antioxidant enzymes such as SOD (r = 0.952***), CAT (r = 0.918***), and POD (r = 0.961***) (**Figure 3**). Similarly, chlorophyll content, Pn, and RWC also exhibited strong intercorrelations (r > 0.95), indicating a coordinated physiological and biochemical adjustment under water deficit condition (**Figure 3**). These interdependent relationships suggested that the accumulation of osmoprotectants and enhanced antioxidant activities directly contributed to CMS and photosynthetic performance during drought stress. Moreover, principal component analysis (PCA) further delineated the underlying variation among genotypes and traits (**Figure 4**). Under drought, tolerant genotypes such as ‘Ajmeri’, ‘DMX4561’, ‘Rawal’, and ‘NARC-21’ aligned positively with vectors corresponding to proline, GB, SOD, CAT, POD, and CMS, indicating strong trait associations and superior adaptive capacity (**Figure 4**). In contrast, susceptible genotypes like ‘Anjasmoro’, ‘Grobogan’, and ‘Dering-1’ grouped on the opposite side of the PCA space, reflecting poor association with these drought-responsive traits and reduced resilience under stress (**Figure 4**). The positioning of tolerant genotypes near the osmolyte and enzyme vectors implies their higher contribution to the total variation explained by principal components, especially under drought.

**Figure 5:**
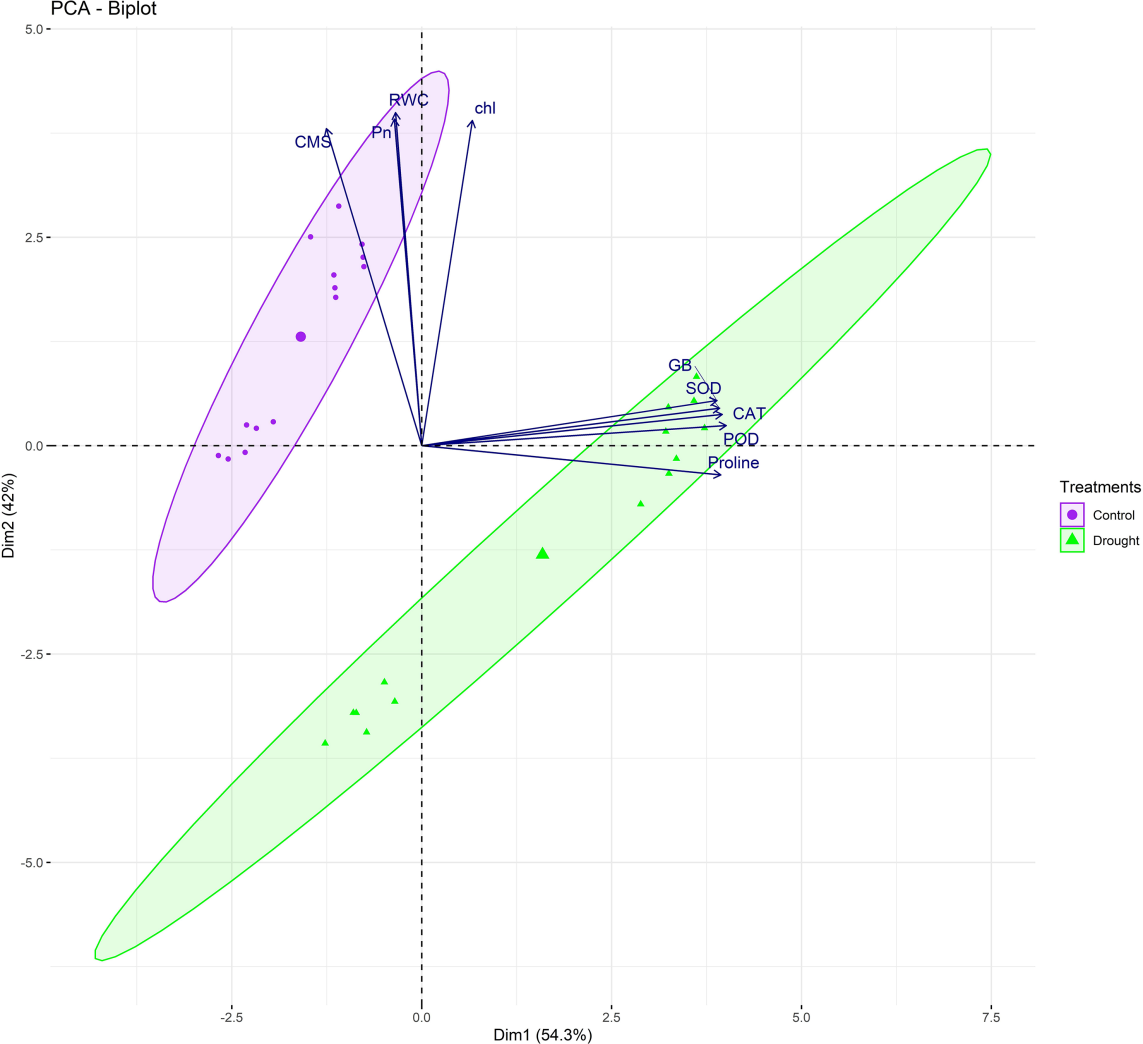
Principal component analysis (PCA) biplots illustrating multivariate relationships among physiological and biochemical traits of drought tolerant (Ajmeri, NARC-21, DMX4561, and Rawal) and drought susceptible (Anjasmoro, Grobogan, and Dering-1) soybean genotypes under control (left) and drought stress (right) conditions. The biplots display genotype scores and trait loadings (vectors), revealing the contribution of individual variables to genotype differentiation. Traits include proline, glycine betaine (GB), superoxide dismutase (SOD), peroxidase (POD), catalase (CAT), chlorophyll content (Chl), photosynthetic rate (Pn), relative water content (RWC), and cell membrane stability (CMS). Genotypes positioned closer to trait vectors indicate stronger positive associations.

Furthermore, the treatment-based PCA biplot illustrated the clear divergence between control and drought conditions (**Figure 5**). Control plants clustered around traits such as chlorophyll, RWC, and Pn, indicating high baseline photosynthetic efficiency and hydration (**Figure 5**). In contrast, drought-stressed genotypes aligned closely with antioxidant enzymes and osmolytes, suggesting that these traits become the major determinants of stress tolerance under water-deficit conditions (**Figure 5**). Notably, the drought cluster was dominated by tolerant genotypes, confirming their shift towards biochemical defense mechanisms when subjected to stress. The shape and spread of ellipses in the biplot further indicate that drought imposes a distinct directional shift in the physiological profile of tolerant genotypes compared to susceptible ones.

**Figure 6:**
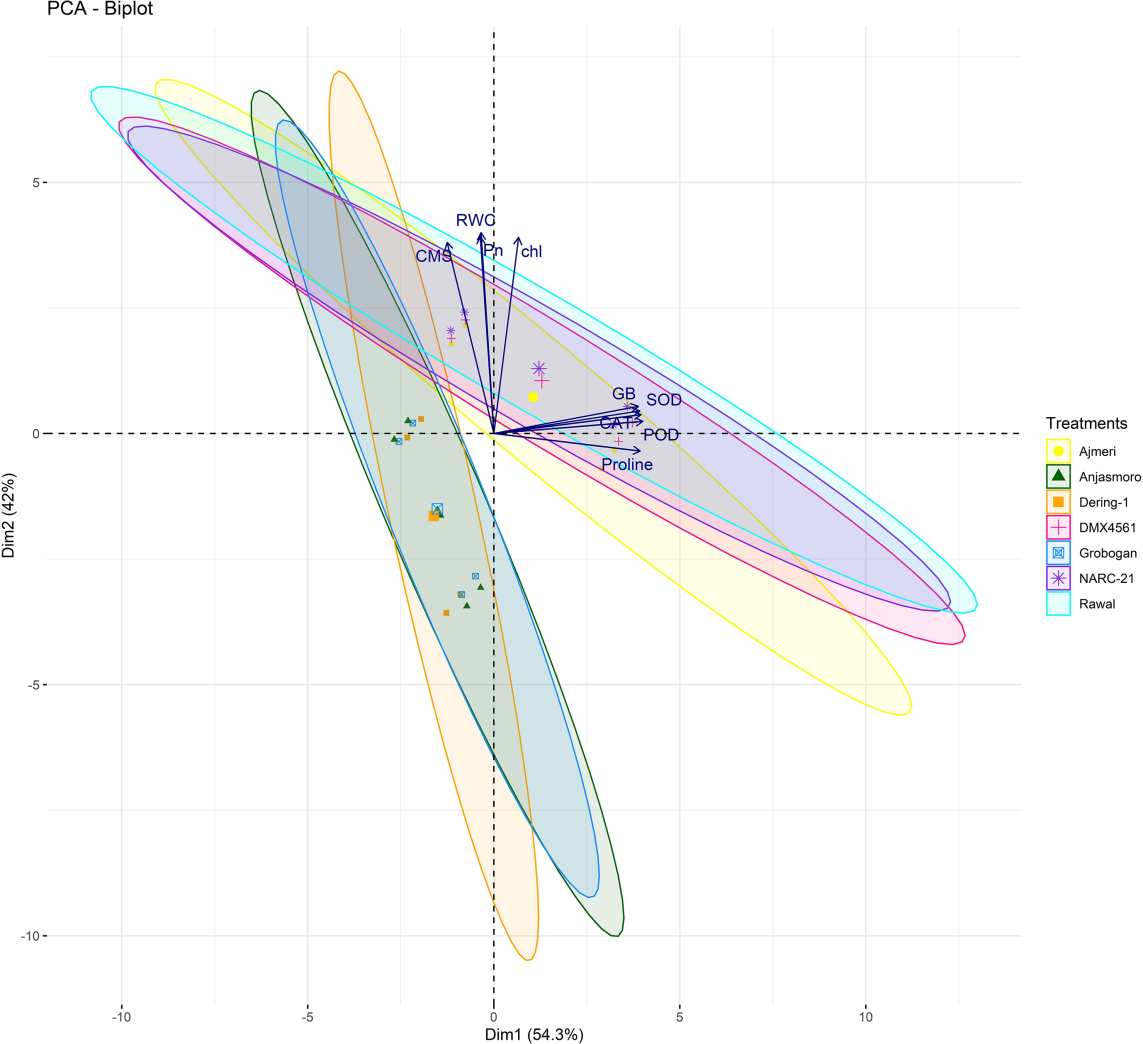
Treatment- and genotype based PCA biplot of soybean cultivars under control and drought stress conditions. The biplot illustrates clustering patterns of drought tolerant (Ajmeri, NARC-21, DMX4561, and Rawal) and drought susceptible (Anjasmoro, Grobogan, and Dering-1) soybean genotypes in response to control and drought treatments. Ellipses represent within-genotype trait variability. Vectors correspond to physiological and biochemical traits, with proline, superoxide dismutase (SOD), peroxidase (POD), catalase (CAT), and glycine betaine (GB) associated with drought tolerant genotypes, whereas chlorophyll content (Chl), and photosynthetic rate (Pn) are more closely associated with control conditions.

Genotype-based PCA showed that tolerant genotypes were grouped in close proximity to key drought-responsive traits, including proline, GB, CAT, and SOD, demonstrating their consistent upregulation (**Figure 6**). On the other hand, susceptible genotypes such as ‘Anjasmoro’ and ‘Grobogan’ occupied peripheral positions in the PCA space and remained distant from stress-related vectors, highlighting their limited adaptability (**Figure 6**). ‘Dering-1’, although slightly closer to the center, still displayed weak associations with protective traits confirming this genotype as drought-sensitive. This genotypic separation provides strong multivariate evidence that drought tolerance in soybean is a function of both osmotic regulation and oxidative defense, with tolerant genotypes excelling in both domains.

**Figure 7:**
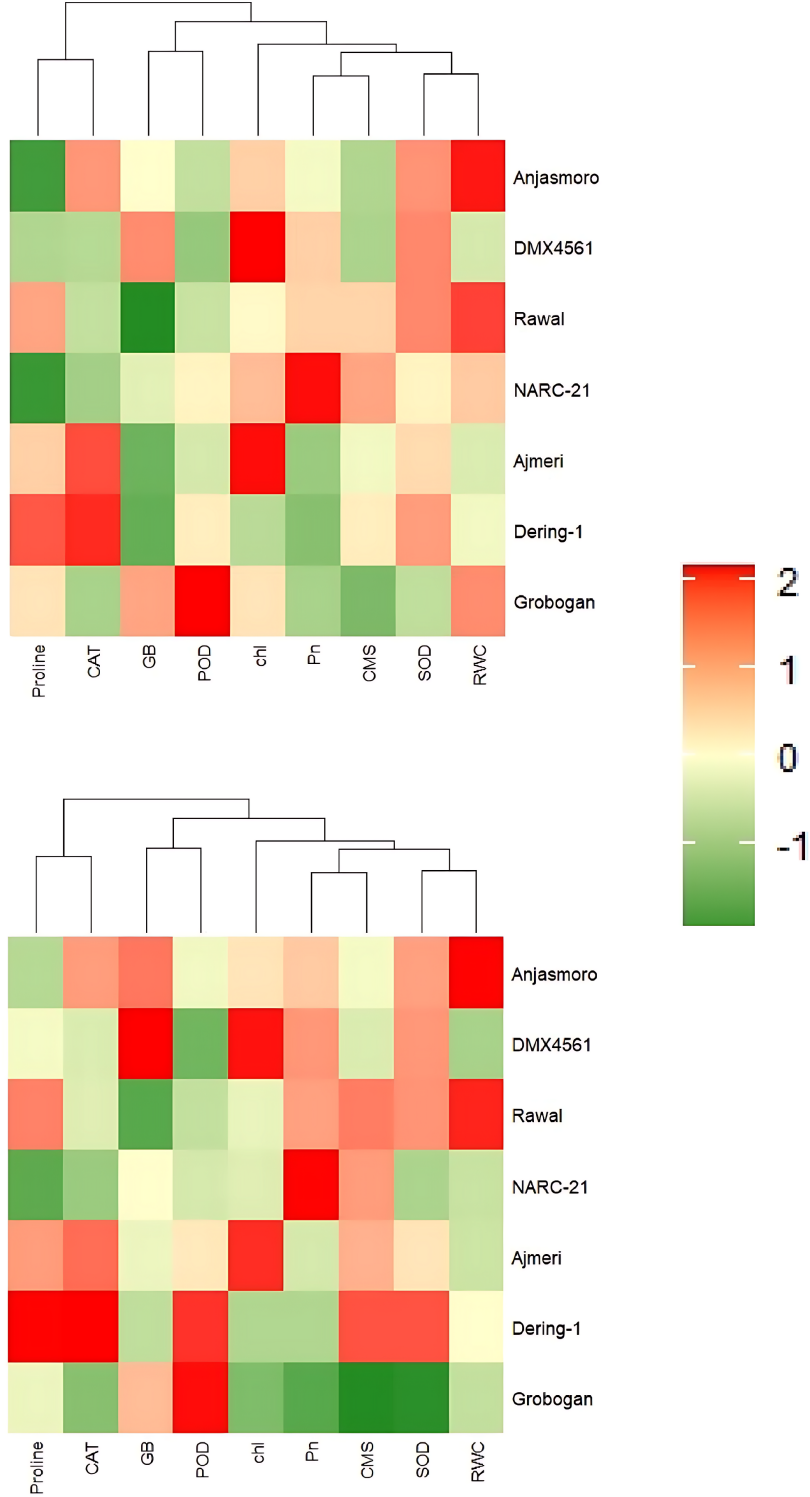
Hierarchical cluster heatmaps of biochemical and physiological traits in soybean genotypes under control (top) and drought (bottom) treatments. The heatmaps illustrate the magnitude and clustering of nine drought responsive traits: proline, catalase (CAT), glycine betaine (GB), peroxidase (POD), chlorophyll content (Chl), photosynthetic rate (Pn), cell membrane stability (CMS), superoxide dismutase (SOD), and relative water content (RWC). Red and green colors indicate higher and lower standardized values, respectively. Dendrograms show genotype clustering based on trait similarity, revealing a distinct separation between drought tolerant (Ajmeri, NARC-21, DMX4561, and Rawal) and drought susceptible (Anjasmoro, Grobogan, and Dering-1) genotypes under drought stress.

The two-way hierarchical clustering presented in **Figure 7** rectified the PCA findings through heatmap visualization of trait intensities across genotypes. Under drought stress, genotypes displayed clear differential responses. **‘**NARC-21’, ‘Rawal’, ‘DMX4561’, and ‘Ajmeri’ generally exhibited higher relative values (orange to red gradients) for several drought-responsive traits, particularly proline, GB, antioxidant enzymes (CAT, POD, SOD), and CMS, although the magnitude of response varied among traits and genotypes (**Figure 7**). In contrast, **‘**Anjasmoro’, ‘Dering-1’, and ‘Grobogan’ were characterized by comparatively lower values (green to yellow gradients) for many traits, most notably CMS, RWC, and antioxidant-related parameters (**Figure 7**). Hierarchical clustering under drought stress resulted in a clearer separation of genotypes, with tolerant genotypes grouping more closely based on their overall physiological and biochemical traits, while susceptible genotypes formed a separate cluster, reflecting contrasting drought response patterns (**Figure 7**).

Together, these statistical analyses provide strong, convergent evidence that drought tolerance in soybean is regulated by a combined network of antioxidant activity, osmoprotectant accumulation, and physiological stability. The close clustering of tolerant genotypes with these stress-mitigating traits across correlation, PCA, and heatmap analyses illustrates their superior resilience and highlights their suitability for drought-prone environments.

## 4 Discussion

The present study provides a comprehensive physiological, biochemical, statistical, and molecular characterization of drought-tolerant and drought-susceptible soybean genotypes. Besides, it revealed that drought tolerance is conferred through coordinated regulation of antioxidant defense, osmotic adjustment, membrane stability, and transcriptional activation of stress-related genes. Across all **Figures 1**-**7**, tolerant genotypes, ‘Ajmeri’, ‘NARC-21’, ‘DMX4561’ and ‘Rawal’ consistently surpassed the susceptible genotypes, ‘Anjasmoro’, ‘Grobogan’ and ‘Dering-1’, confirming that multiple, interlinked mechanisms impart superior drought resilience in soybean.

The biochemical evaluation f (**Figure 1**) show that drought tolerance is strongly associated with enhanced antioxidant enzyme activity. Tolerant soybean genotypes recorded higher catalase (CAT), superoxide dismutase (SOD), and peroxidase (POD) activities, enabling more efficient detoxification of reactive oxygen species (ROS) generated under drought stress. This agrees with past studies reporting that drought-tolerant soybean cultivars sustain higher antioxidant capacity to limit oxidative damage (Liu et al., 2017; Hasanuzzaman et al., 2020; Alzahrani, 2024). In present study, the enhanced enzymatic activity was paralleled at the transcriptional level (**Figure 2**), where *GmSOD1*, *GmPOD1*, and *GmCAT1* showed greater fold induction in tolerant genotypes (~2.7–3.5-fold) compared to susceptible genotypes (~1.6–2.0-fold). Similar findings were reported by Alzahrani (2024) and Wang et al. (2025), suggesting that antioxidant enzyme upregulation is a vital component of soybean drought tolerance.

Similarly, physiological comparison (**Figure 1**) demonstrated that tolerant soybean genotypes maintained higher chlorophyll content, Pn, and CMS than susceptible ones. This explicates that photoprotective mechanisms and structural membrane integrity are stronger in tolerant genotypes. These results align with Hossain et al. (2014) and Zhou et al. (2024), who reported that drought-tolerant soybean cultivars sustain chlorophyll and Pn through delayed senescence and improved photosystem stability. Higher CMS in tolerant soybean genotypes also indicates reduced lipid peroxidation, likely due to ROS scavenging, as reported by Mafakheri et al. (2010) and Lu et al. (2024). Besides, the biochemical osmotic adjustment was a key differentiator as shown in **Figure 1**, where tolerant genotypes accumulated significantly higher proline and GB, maintaining RWC above 80% under drought. This pattern corresponded with higher expression of osmolyte biosynthesis genes *GmP5CS* and *GmBADH2* as shown in **Figure 2**. Previous studies by Soleimani et al. (2015), Liu et al. (2017), and Golestan Hashemi et al. (2018) concluded that osmolytes stabilize proteins, protect membranes, and maintain turgor, thus enhancing drought tolerance. The positive relationship between osmolyte levels and RWC was further rectified from the findings of Alzahrani (2024).

Moreover, the statistical analyses (**Figures 3**-**7**) further validated these physiological and biochemical trends. Strong positive correlations between proline, GB, antioxidant enzymes, and RWC proves their synergistic role in stress mitigation, in agreement with Alzahrani (2024). PCA based separation of tolerant and susceptible soybean genotypes under drought (**Figures 4**-**6**) supports earlier reports by Alzahrani (2024) and Ding et al. (2024) that multivariate trait integration can effectively discriminate drought tolerance levels. The two-way hierarchical clustering in **Figure 7** reinforced this grouping, revealing that tolerant genotypes share a high-intensity trait profile under stress, as reported by Guzzo et al. (2021).

The gene expression data in **Figure 2** provide molecular confirmation of these adaptive mechanisms. Tolerant genotypes exhibited higher accumulation of the transcription factor *GmDREB2* (~2.0–3.3-fold), dehydration-related protein *GmLEA-D11* (~2.8–3.6-fold), and osmolyte biosynthesis genes *GmP5CS* and *GmBADH2*, suggesting stronger stress signaling and osmotic regulation. These results were in agreement with those of Pham et al. (2020) and Savitri (2023), who reported that overexpression of *DREB* and *LEA* genes induce drought tolerance via downstream protective pathways. Moreover, the maintenance of aquaporin gene, *GmPIP2;9* expression in tolerant genotypes was consistent with the findings of Lu et al. (2018) that aquaporins sustain water flow and contribute to RWC stability under water deficit conditions. The smaller decline in chlorophyll biosynthesis gene *GmCHLG* in tolerant genotypes is consistent with Zhang et al. (2018) who reported that tolerant cultivars maintain chlorophyll synthesis capacity during water stress.

Overall, this study confirmed that drought tolerance in soybean is not imparted by a single trait but results from an integrated network of biochemical, physiological, and transcriptional adjustments. Tolerant genotypes demonstrated: (1) Robust antioxidant enzymes activity, reducing oxidative damage; (2) Osmotic adjustment through proline and GB accumulation, retaining cell turgor; (3) Structural integrity of membranes and photosynthetic apparatus; (4) Coordinated regulation of stress related genes activating stress-responsive pathways; and (5) Trait synergistic association evident in statistical clustering.

## 5 Conclusion

The physiological, biochemical, statistical, and molecular evaluations illustrated in this study provided a comprehensive and practically relevant framework for improving drought tolerance in soybean. The consistently superior performance of ‘Ajmeri’, ‘NARC-21’, ‘DMX4561’, and ‘Rawal’ across multiple drought-responsive traits identified these genotypes as valuable donor parents for use in conventional and molecular breeding programs targeting water-limited environments. Traits such as RWC, CMS, osmolyte accumulation (proline and GB), antioxidant enzyme activity, and the expression of drought-responsive genes emerged as reliable selection indicators, which can be effectively exploited for early-generation screening under controlled or field-based drought conditions. The strong association between physiological performance and molecular responses further supports the integration of these traits into high-throughput phenotyping and selection pipelines, enabling more precise identification of drought-tolerant lines. Collectively, these findings facilitate the translation of drought tolerance mechanisms or adaptations into applied breeding methods and provide genotype-specific insights that can accelerate the development of climate-resilient soybean cultivars for arid and semi-arid regions across the globe.

## Data Availability

The original contributions presented in the study are included in the article/supplementary material. Further inquiries can be directed to the corresponding author.
